# Supplementary figures and images for: Protein phosphatase-1 inhibitor-2 promotes PP1γ positive regulation of synaptic transmission
Source: Front Synaptic Neurosci. 2022 Oct 6;14:1021832. doi: 10.3389/fnsyn.2022.1021832 (PMC9582336; doi:10.3389/fnsyn.2022.1021832)

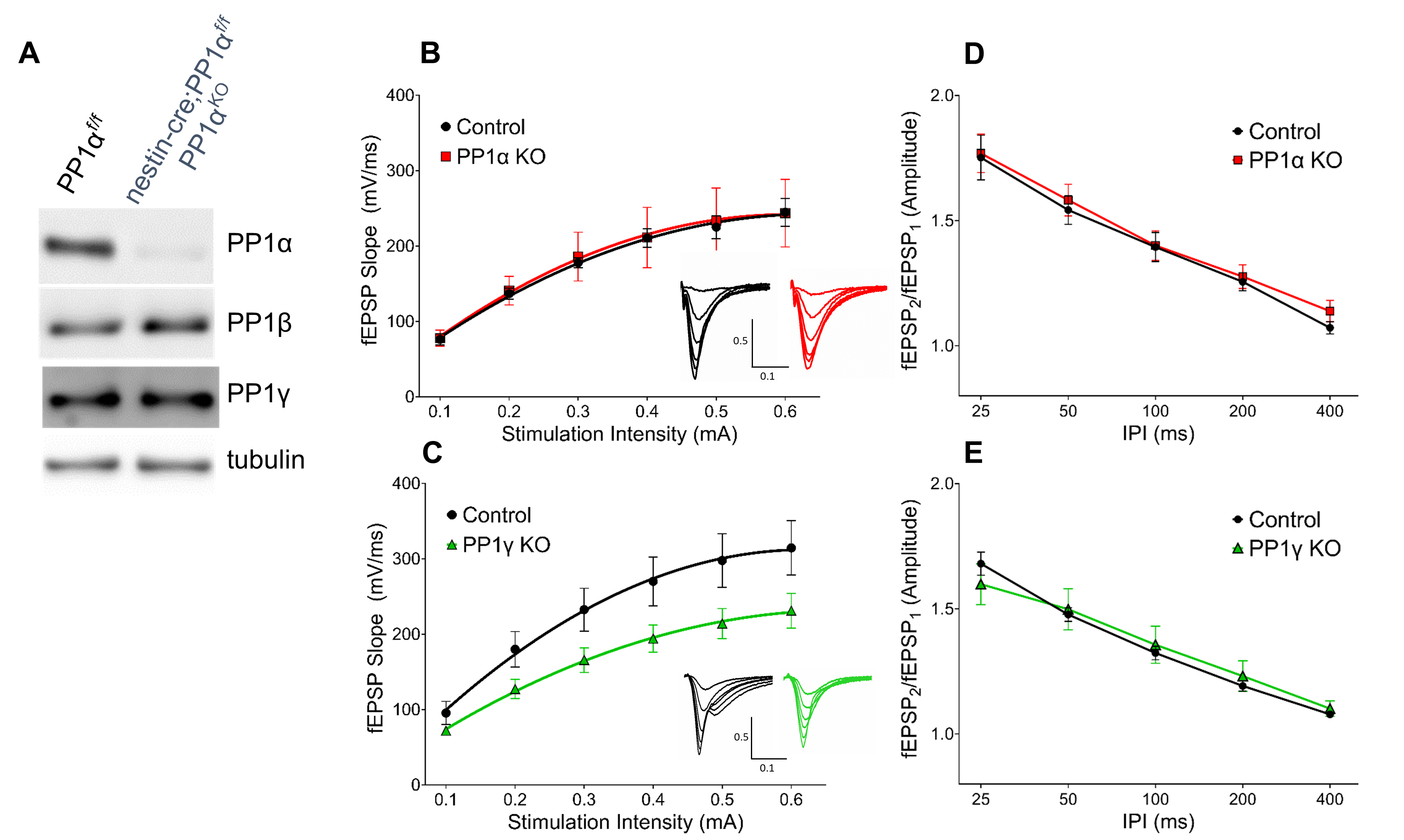

Supplement: Supplementary Figure 1 — PP1α does not play a role in synaptic transmission. (A) Successful knockout of PP1α protein in nestin-cre;PP1αf/f mice. (B–E) Results from field recordings in acute hippocampal slices at Sch-CA1 synapses. (B,C) There is no difference in basal synaptic transmission in PP1αKO mice, but a significant decrease in PP1γKO mice [two-way RM-ANOVAs, genotype: F(1, 10) = 0.02, p = 0.899; F(1, 14) = 4.79, p < 0.05, respectively). (D,E) There is no change in paired pulse facilitation (PPF) at the Sch-CA1 pyramidal synapses in PP1αKO or PP1γKO mice [two-way RM-ANOVAs, genotype: F(1, 10) = 0.172, p = 0.687; F(1, 14) = 0.006, p = 0.938, respectively). Data are from the following number of mice/slices: (B), control 2/7, knockout 2/5; (C), control 3/7, knockout 3/9; (D), control 2/6, knockout 2/6; (E), control 3/6, knockout 3/10. [file Image_1.tiff]
